# Machine learning accurately predicts food exchange list and the exchangeable portion

**DOI:** 10.3389/fnut.2023.1231873

**Published:** 2023-08-10

**Authors:** David Jovani Hernández-Hernández, Ana Bertha Perez-Lizaur, Berenice Palacios-González, Gesuri Morales-Luna

**Affiliations:** ^1^Departamento de Física y Matemáticas, Universidad Iberoamericana Ciudad de México, Ciudad de México, Mexico; ^2^Departamento de Salud, Universidad Iberoamericana Ciudad de México, Ciudad de México, Mexico; ^3^Laboratorio de Envejecimiento Saludable, Centro de Investigación Sobre Envejecimiento (CIE-CINVESTAV Sur), Instituto Nacional de Medicina Genómica, Ciudad de México, Mexico

**Keywords:** artificial neural networks, food classification, exchangeable portion, nutrient composition, equivalent portion

## Abstract

**Introduction:**

Food Exchange Lists (FELs) are a user-friendly tool developed to help individuals aid healthy eating habits and follow a specific diet plan. Given the rapidly increasing number of new products or access to new foods, one of the biggest challenges for FELs is being outdated. Supervised machine learning algorithms could be a tool that facilitates this process and allows for updated FELs—the present study aimed to generate an algorithm to predict food classification and calculate the equivalent portion.

**Methods:**

Data mining techniques were used to generate the algorithm, which consists of processing and analyzing the information to find patterns, trends, or repetitive rules that explain the behavior of the data in a food database after performing this task. It was decided to approach the problem from a vector formulation (through 9 nutrient dimensions) that led to proposals for classifiers such as Spherical K-Means (SKM), and by developing this idea, it was possible to smooth the limits of the classifier with the help of a Multilayer Perceptron (MLP) which were compared with two other algorithms of machine learning, these being Random Forest and XGBoost.

**Results:**

The algorithm proposed in this study could classify and calculate the equivalent portion of a single or a list of foods. The algorithm allows the categorization of more than one thousand foods with a confidence level of 97% at the first three places. Also, the algorithm indicates which foods exceed the limits established in sodium, sugar, and/or fat content and show their equivalents.

**Discussion:**

Accurate and robust FELs could improve implementation and adherence to the recommended diet. Compared with manual categorization and calculation, machine learning approaches have several advantages. Machine learning reduces the time needed for manual food categorization and equivalent portion calculation of many food products. Since it is possible to access food composition databases of various populations, our algorithm could be adapted and applied in other databases, offering an even greater diversity of regional products and foods. In conclusion, machine learning is a promising method for automation in generating FELs. This study provides evidence of a large-scale, accurate real-time processing algorithm that can be useful for designing meal plans tailored to the foods consumed by the population. Our model allowed us not only to distinguish and classify foods within a group or subgroup but also to perform the calculation of an equivalent food. As a neural network, this model could be trained with other food bases and thus improve its predictive capacity. Although the performance of the SKM model was lower compared to other types of classifiers, our model allows selecting an equivalent food not from a group previously classified by machine learning but with a fully interpretable algorithm such as cosine similarity for comparing food.

## Highlights

- ML is a method for automation to generate FELs.
- ML algorithm allows correct food categorization to FELs.
- ML algorithm reduces the time needed for food categorization.
- ML algorithm allows the calculation of an equivalent food.

## 1. Introduction

The Food Exchange List (FEL) arises from the need to offer a simple didactic tool to give food variety to the individual diet of patients with type 2 diabetes (T2D) (1). Because of their usefulness, practitioners in nutrition and dietetics have been using them in menu planning and nutritional education of patients, especially those with metabolic diseases such as diabetes, obesity, and cancer, among others (2). The first edition was published in 1950 and was developed by the American Dietetic Association, the American Diabetes Association, and the United States Public Health Service (3). In Mexico, it began to be used in the 1970s as a translation of the American list. In 1988, the FEL was adapted to include typical foods of the culinary and gastronomic customs of the Mexican population [Mexican System of Equivalent Foods (SMAE)] (4, 5).

The SMAE is based on the grouping of foods proposed in the Mexican Official Standard “NOM-043-SSA2-2012, Basic health services. Food safety promotion and education. Criteria for the provision of guidance” and the concept of “Equivalent Food” (6, 7). An “Equivalent Food” is a portion that approximately contributes the same macronutrient (energy, protein, carbohydrate, and fat) value to those of its same group of foods in quality and quantity, which allows them to be interchangeable without significant differences in dietary intakes of patients (8, 9). The SMAE is grouped into the usual exchange categories, but some subgroups are proposed based on the secondary macronutrient contents (e.g., different sugar, fat, and protein amounts) (5).

Given the changes in the population’s eating patterns, changes in the food marketplace, the permanent innovation of new products, and globalization, it is considered a latent need to analyze and update the FELs, verify the information, and add greater detail to the foods included (10, 11). Distinguishing and classifying food within a group or subgroup to finally offer the user the equivalent portion can be a challenging and resource-intensive task since it is, at this time, a manual process, albeit necessary for a better understanding of foods and diets.

Machine learning (ML) has become dominant, especially when text datasets are on large scales, such as in computer science, medical science, healthcare, and agriculture (12, 13). The typical description of these methods is that they exploit the amount of data available due to their ability to model non-linear relationships and high-level interactions (14, 15). Neural Networks are among the most powerful (and popular) algorithms used for classification. They take inputs as vectors, perform some computations, and then produce an output vector. The output vector is then compared with the ground truth labels, and the weights are tweaked (i.e., trained) to yield better results. To train the Neural Network, we feed our input data in feature vectors representing the data’s important gist. Neural networks (NNs) computing systems allow computers to learn from experience and understand the world through a hierarchy of concepts, each defined by its relation to more straightforward concepts (16). By gathering knowledge from experience, this approach avoids the need for scientists to specify all the knowledge and rules that the computers previously needed. The hierarchy of concepts enables the computer to learn complicated concepts by building them out of simpler ones (17).

Most studies on classifying foods using deep learning employ pictures, images, spectroscopic, hyperspectral signals, and mass spectrometry data (18–22). An ML approach has also been used to predict added sugar and fiber content using nutrient information packages (23, 24). Recently, Ma et al. achieved up to 99% accuracy for food classification and 0.93 ~ 0.97 for calories, protein, sodium, carbohydrate, and lipids estimation using a generic deep-learning-based technique (25).

The present study aimed to generate an algorithm using artificial neural networks and how we arrived at it to predict the classification of foods, calculate the equivalent portion for the most similar foods based on cosine similarity, and indicate foods with high sodium, sugar, or fat content.

## 2. Materials and methods

### 2.1. Dataset

The Mexican food exchange list had six phases:

1. Obtain the nutritional composition per 100 g from food composition databases (26, 27).

2. Calculate the portion of foods using household measurements (240 mL cup, 15 mL tablespoon, 5 mL teaspoon, 16 tablespoon cup, three teaspoon tablespoon, medium piece).

3. Classification of foods into groups and subgroups.

4. Definition of the key nutritional component and its quantity for each group (Table 1).

**Table 1:** Determination of equivalent portion based on the reference foods.

Groups	Reference food	Portion	Key nutritional component		
			Protein (g)	Fat (g)	Carbohydrates (g)
Vegetables	Carrot	½ cup			4
Fruits	Apple	One piece			15
Cereals and by-products	Tortilla	One piece			15
Legumes	Cooked beans	½ cup	8		
Meats	Egg	One piece	7		
Milk	Milk	One cup	9		12
Fat and oils	Oil	One teaspoon		5	
Sugar, honey, and candy	Sugar	2 teaspoons			10

5. Calculate the food portions of each sub-group in weight (g) and household measures like cups and others.

The portion needs to be adequate to obtain the value of the key nutritional component (on average ± 2 standard deviations) and reasonable for use as a household measure: rounding the weight in grams to the nearest five or zero for high-moisture foods such as fruits, vegetables, animal foods, cooked cereals, and cooked legumes.

The weight is indicated in grams for dry or raw foods such as cereals and legumes without rounding (6). Classification of foods in other sub-groups according to the content of other nutritional components, for example, cereals, meats, and milk with different fat content; milk with high sugar content; oils and fats with different protein content and sugars with different gauze content in Table 2, nine variables are presented, including the macronutrients, which are contained in most food categories. Also, the units are included in this table, being all the variables positive scalars. Is important to mention that the total amount of data with which the study was carried out was 2,877 data. Using 2,201 data for training and 576 data for testing, which corresponds to 80 and 20%, respectively. In the case of the XGBoost algorithm 50% was used of the whole dataset for training, 25% for the testing, to find the best parameters and the rest, 25%, used for the validation.

**Table 2:** Variables considered for the code.

Variable	Units
Energy	kcal
Protein	g
Total lipids	g
Saturated fat	g
Cholesterol	mg
Carbohydrates	g
Sugar	g
Fiber	g
Sodium	mg

An important consideration regarding the code is that variables such as sodium and cholesterol were not defined in the database for some foods because measurements of these two variables were irrelevant to the nutrition label of those foods: therefore, this equivalent to filling in the missing data with zero.

At the algorithm input, the carbohydrate and lipid variables were broken into more nutrients, respectively:


(1) Carbohydrates=Startch+Sugar+Fiber,

and


(2) Totallipids=Usaturatedfat+Saturatedfat.

## 3. Dimensionless

The first step in considering the input data was to dimensionless it as follows:


(3) $$\bar{x_{ij}}=\left(N*x_{ij}\right){\left(\overset{N}{\underset{i=1}{\sum }}x_{ij}\right)}^{-1}\;$$

Where N is the number of the foods and $x_{ij}$ is the nutrient j of the food i .

### 3.1. Similarity and equivalence factor

After dimensionless, normalization is one of the fundamental processes before running the code. It is already known that the nutrient of the database is referred to as 100 g, but this does not mean that foods should be immediately compared with each other, and a mean square error is not enough for that. It is necessary to build equivalent foods, starting from the following rule:


(4) $$\vec{A}=\alpha \vec{B}.$$

Where $\vec{A}$ and $\vec{B}$ are food vectors in the space of nutrients, where each of the nutrients considered is one dimension, α is the optimal equivalence factor that multiplies the components of $\vec{B}$ , bringing the food as close as possible to $\vec{A}$ according to the definition of an equivalent food.

Given the vector origin of the food, the following equivalence function that satisfied the properties mentioned before is proposed by the standard household similarity measure.


(5) $$Sim\left(x,y\right)=Sim_{cosine\left(x,y\right)}=\frac{\langle x,y\rangle }{{\left\|x\right\|}_{2}\cdot {\left\|y\right\|}_{2}}=\frac{\sum_{i}x_{i}y_{i}}{\sqrt{\sum_{i}x_{i}^{2}}\cdot \sqrt{\sum_{i}y_{i}^{2}}}=\cos\theta ,$$

Is given by,


(6) $$Sim\left(A,B\right)=\langle \vec{A},\vec{B}\rangle =\frac{\vec{A}\cdot \vec{B}}{\left|\vec{A}\right|\left|\vec{B}\right|}=\cos\theta_{AB}.$$

The similitude between A and B is equal to the projection of the unit vector of B on the unit vector A . Thus, the formulation of the similarity between foods was based on the nutrient concentration and not on distance, as presented in other works (25). This formula has advantages since it does not depend on the amount of both foods but on the concentration; the amount needed to scale from food B to A in terms of units of B is defined as follows:


(7) $$\alpha =\frac{\left|\vec{A}\right|\cos\theta_{AB}}{\left|\vec{B}\right|}.$$

The most optimal scalar is calculated in the dimensionless space, and this does not imply that it is the same, nor that it leads to the closest point of food A in the nutrient space since having different units causes distributions in different ranges, as an example, a difference of 50 kcal has not the same impact to the consumer as a difference of 50 g of saturated fat for a 100-gram food.

### 3.2. Classification

Once the tool has been created to compare the similarity between foods, the representative centroids of each food group were placed using the Spherical K-means (SKM) algorithm which is the most common algorithm of aggrupation that is used in this kind of analysis (28). This algorithm has been studied for many decades, and the main objective of SKM is to minimize the differences between each group and maximize the differences between clusters. The following formula gives the classification:


(8) $$Mostlikelygroup=argmax\left[Sim\left(A,W_{j}\right)\right].$$

Where $W_{j}$ is the centroid which represents the j− category.

**Figure 1:**
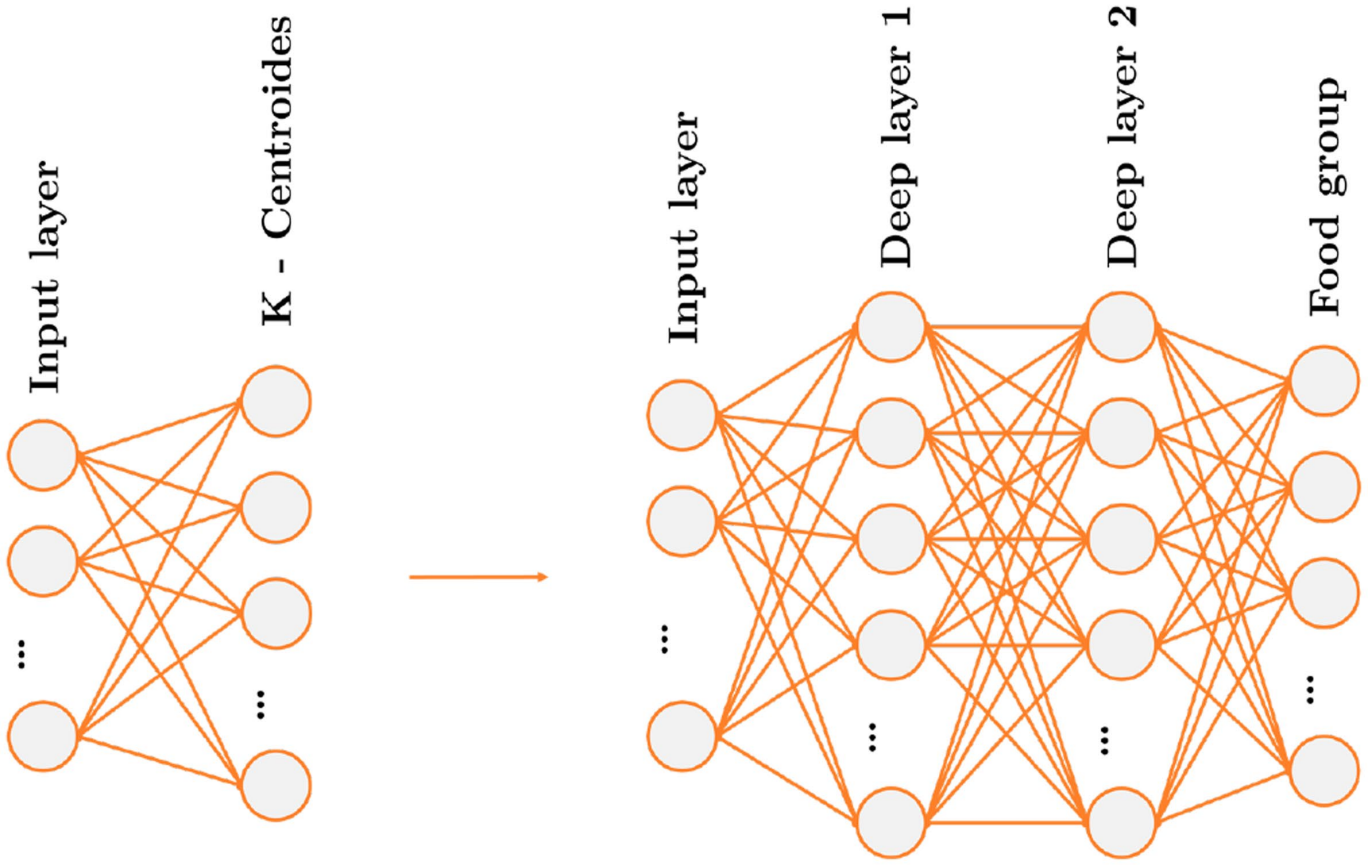
Map of the Spherical K-means algorithm represented in nodes and the map of the neural networks.

The above is analog to a single layer neural network with no activation function and with the proviso that the weights will be normalized, so you can remove all these constraints and treat it as a multilayer perceptron (MLP) neural network, see Figure 1.

**Figure 2:**
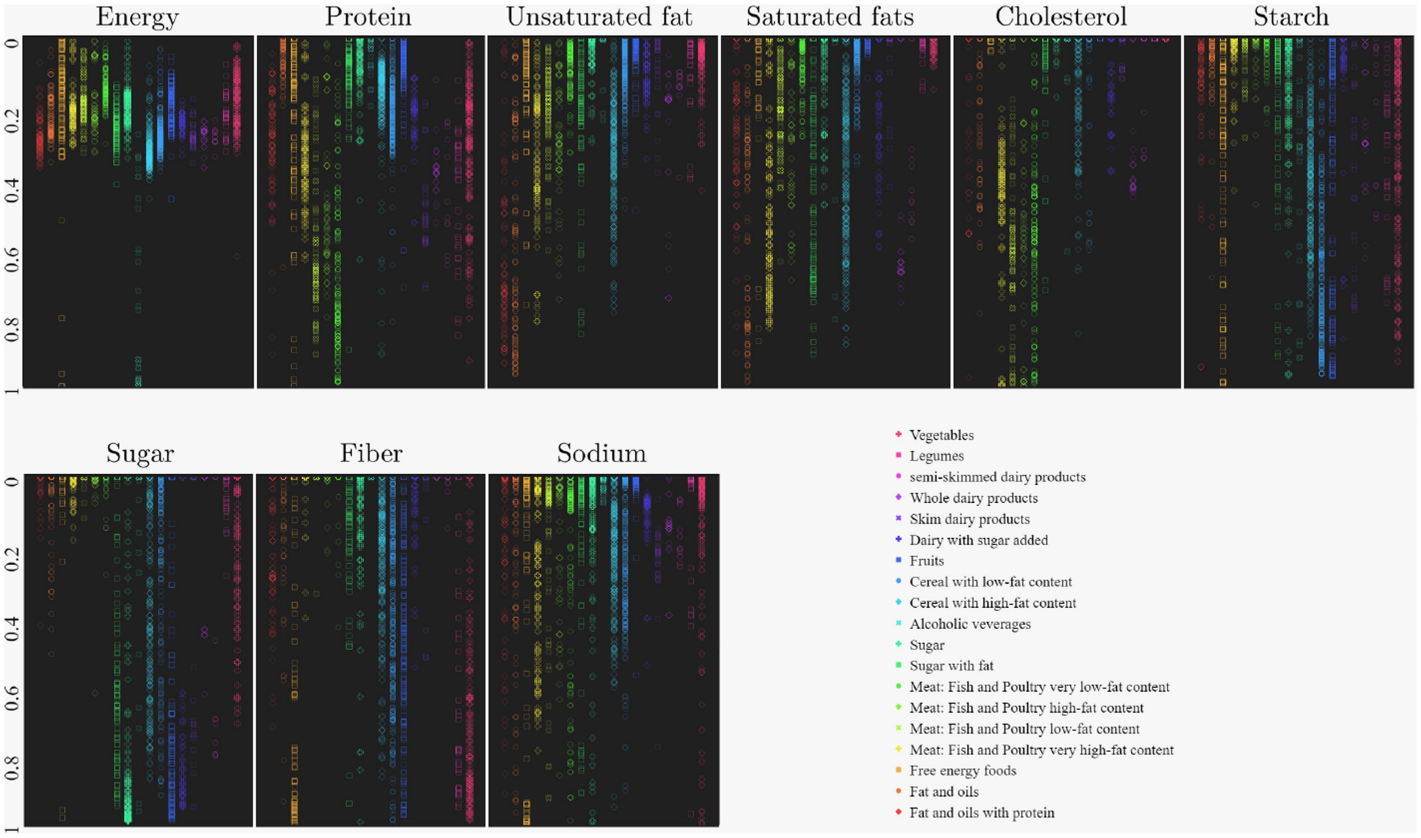
Color map of the distribution of nourishment components for color segmentation of the different food groups.

As a result of this process and the normalization of the input data, Figure 2 was obtained, where the distributions of the nourishment components are displayed given the segmentation proposed by SMAE. This result was also the input for the training of the MLP.

Figure 2 shows that each nutrition group has a well-defined and different distribution for each nutritional component, indicating a good segmentation. For example, in the sugar nutrient, the sugars groups with and without fat stand out for the high concentration of their distribution, the same for protein, where the foods of animal origin stand out, mainly Meat: Fish and Poultry have very low-fat content. Some information was lost during the dimensionless food vector, so its magnitude was introduced as a new variable in the input vector that could help the neural network classify; otherwise, it will be discarded during the learning process.

The results of MLP model have been compared with two algorithms of machine learning to evaluate which of multiple options can be used to obtain better results; these two algorithms are Random Forest and XGBoost. On the one hand, Random Forest, several numbers of estimators, called trees, were tried. Also, different maximum tree depth was tried, which is a limit to stop the splitting of nodes when the specified tree depth has been reached during the creation of the initial decision tree. On the other hand, For XGBoost, a search for the best parameters was performed using the test dataset.

### 3.3. Model

**Figure 3:**
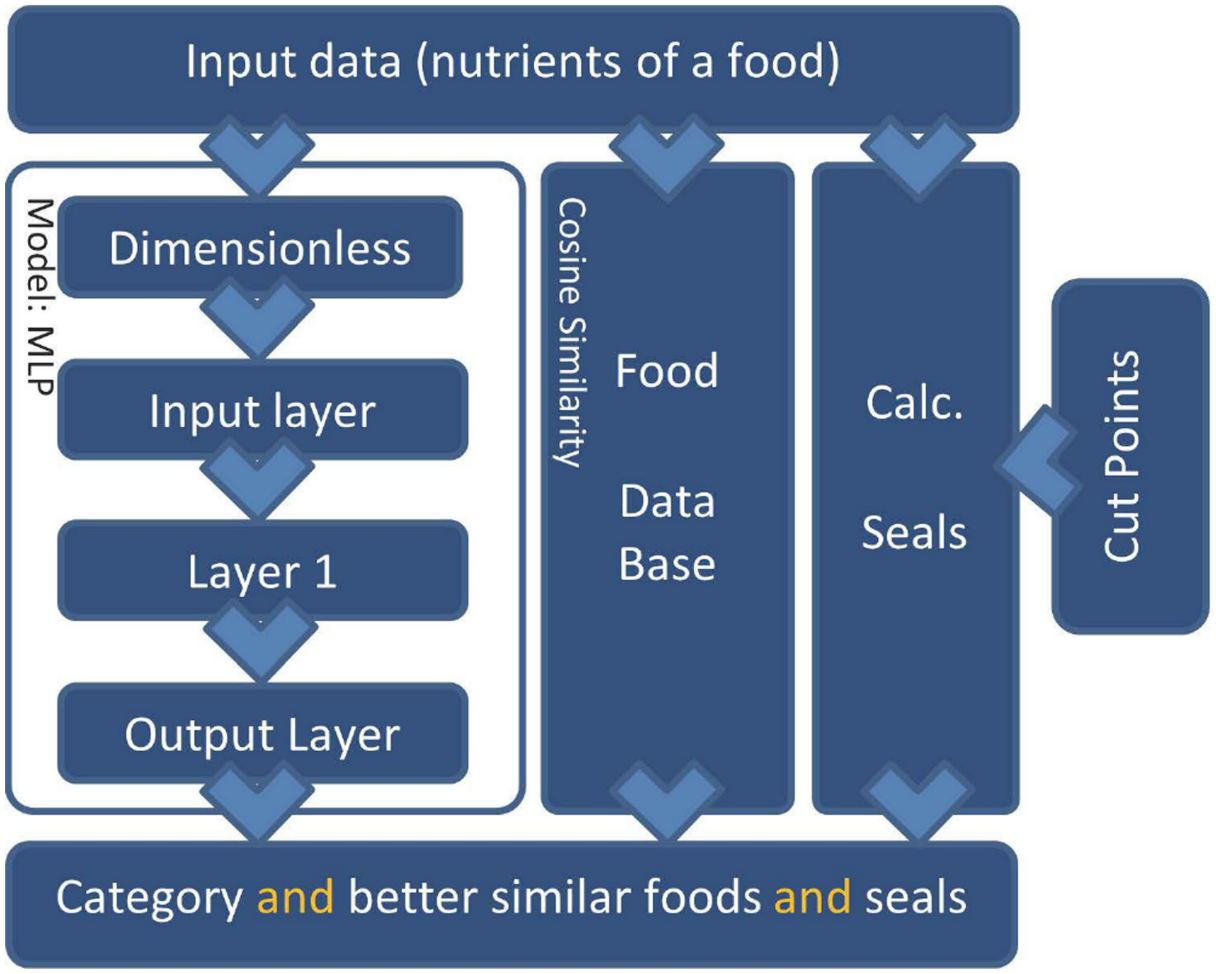
Diagram of the MLP model, being layer 1 and 2 the hidden layers of the neural network. For the SKM layer 1 and 2 were eliminated and the transfer functions were modified between each layer.

Once data treatment, the MLP model was selected according to the conditioning of the variables; the chosen approach was SKM and MLP for interpretability purposes (25) and several topologies were tried to build the MLP model (Figure 3).

### 3.4. Cut points

Additional to the prediction of the nutritional group of each food, a function was added to compute the cut points of the sodium, sugar and/or fat food content (Table 3), using the methodology according to the Mexican Official Standard, NOM-051-SCFI/SSA1-2010 (29).

**Table 3:** Cut point for labeling.

	Energy	Sugar	Saturated fat	Trans fat	Sodium
Solids in 100 g of product	≥275 total of kcal	≥10% of the total of energy from free sugars	≥10% of the total of energy from saturated fat	≥1% of the total of energy from trans fat	≥1mg of sodium per kcal or ≥300mg calorie free drinks: ≥45mg of sodium
Liquids in 100 mL of product	≥70 total of kcal or ≥8 free sugar				
Label	Excess calories	Excess sugars	Excess saturated fat	Excess trans fat	Excess sodium

## 4. Results

**Figure 4:**
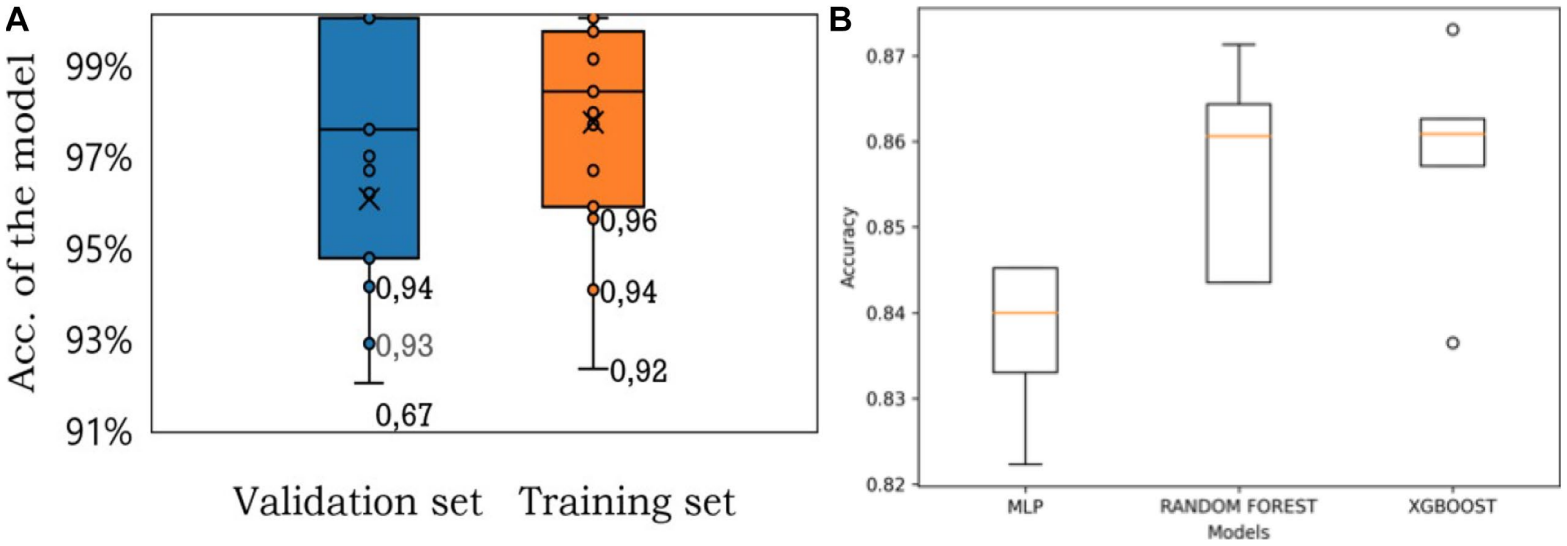
**(A)** Accuracy distribution of the MLP model for training and validation sets for all 19 categories. **(B)** The comparison between each model.

To evaluate the performance of different algorithms, three models were tested using stratified cross-validation with 5 splits. The algorithms under consideration were MLP, Random Forest, and XGBoost. By employing this validation technique, the models were assessed and compared based on their respective accuracy percentages (Figure 4B). The most important thing is accuracy that the three algorithms bring which one could be more useful for this kind of study. The mean accuracy for the algorithms is reported in Table 4.

**Table 4:** Average accuracy of the models.

Model	Accuracy (%)
MLP	84.01
Random forest	86.03
XGBoost	86.12

Upon analyzing these results, it is evident that both Random Forest and XGBoost exhibit higher performances compared to MLP. The Random Forest algorithm achieves an accuracy of 86.03%, while XGBoost surpasses it slightly with an accuracy of 86.12%. These findings suggest that both Random Forest and XGBoost are more effective in addressing the problem at hand, outperforming MLP in terms of accuracy.

It is important to note that while XGBoost and Random Forest showcase similar accuracy levels, the slight advantage of XGBoost implies that it may be a preferable choice when accuracy is the primary evaluation metric. However, further analysis is required to determine if the difference in accuracy between these two models is statistically significant.

These updated results highlight the potential of ensemble-based methods, such as Random Forest and XGBoost, in achieving higher accuracies in this type of problem. It is worth noting that the specific characteristics and requirements of the dataset should also be considered when selecting the most suitable algorithm for practical applications.

**Figure 5:**
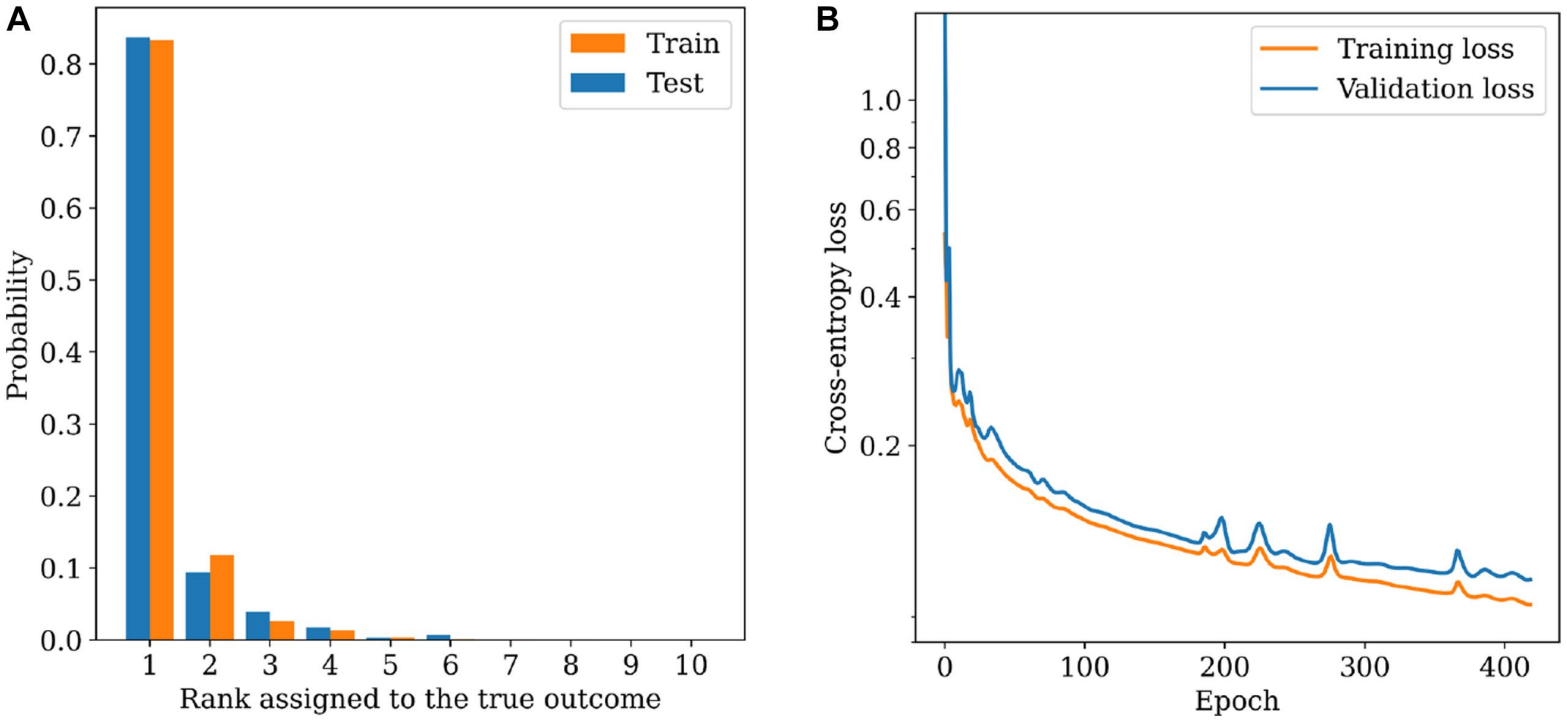
MLP Model **(A)** assigned place of the true group. **(B)** Loss of the training and validation datasets.

For the MLP model was necessary to observe the model’s performance during the training phase (Figure 5B). And get the graph of the assigned place of the valid group (Figure 5A).

The prediction model (MLP) for the food category was trained with 80% of the database, which considers 2,877 registers of food of different categories. This model gets 97.83% accuracy during the training. Meanwhile, during the validation process, we get 97.05% accuracy (Table 5).

**Table 5:** Accuracy of the model considering the n− first places.

Rank assigned to the true outcome	Cumulative training accuracy at the place (%)	Cumulative accuracy at the place (%)
1	83.36	83.68
2	95.18	93.06
3	97.83	97.05

Regarding the food classification (Table 6), algorithm performance is shown for each category of foods and their size during the training and validation process.

**Table 6:** Accuracy of the training and validation set of the model for each food group.

Food category	Accuracy training (%)	Training foods	Accuracy validation (%)	Validation foods
Oils and fats with protein	95.74	94	94.12	17
Fat and oils	98.39	124	100.00	28
Free energy foods	95.85	193	94.74	38
Meat: Fish and poultry very high-fat content	99.17	120	100.00	30
Meat: Fish and poultry low-fat content	99.11	112	96.97	33
Meat: Fish and poultry high-fat content	96.67	60	100.00	17
Meat: Fish and poultry very low-fat content	98.42	190	100.00	52
Sugar with fat	95.60	91	96.15	26
Sugar	97.66	214	96.83	63
Alcoholic beverage	100.00	29	100.00	6
Cereal with high-fat content	99.23	260	100.00	69
Cereal with low-fat content	99.71	349	97.56	82
Fruits	97.92	192	92.00	50
Dairy with sugar added	92.31	39	92.86	14
Skim dairy products	100.00	19	100.00	7
Whole dairy products	100.00	25	100.00	5
Semi-skimmed dairy products	100.00	9	100.00	3
Legumes	96.67	30	66.67	6
Vegetables	94.04	151	96.67	30

In Figure 4A, the distribution precision of the algorithm for the training and validation sets is shown in Figure 4B, the accuracy of three algorithms is shown being Random Forest and XGBoost the best algorithms to deal with this case presented in this manuscript.

## 5. Discussion

According to statistics, 671 million and 439 million of the global population suffer from obesity and T2D, respectively (30). Who also estimated that 4.2 million deaths were related to these diseases, expecting the numbers to increase constantly. Many cross-sectional, prospective, and retrospective studies have found significant associations between nutrients, foods, and dietary patterns in preventing and managing T2D and obesity (31). Thus, innovative healthcare for them is rapidly drawing public attention. For instance, those sensitive to food intake and weight changes may want to keep track of the calories and amounts of carbohydrates, proteins, fats, and other nutrients (20). One approach used to provide that information in a simple and accessible way is by FELs (32). However, distinguishing and classifying food within a group and later calculating the equivalent portion can be challenging and resource-intensive since it is manual. Besides the growing number of food composition datasets and the changes in the eating patterns of the population and the permanent appearance of new foods, it is considered a latent need to analyze and update the FELs, adding more detail about the foods included (33). The preceding makes evident the need for advanced mathematical analysis in this field, which classifies food and offers us the equivalent portion (34). Thus, a methodology that is accurate for classification and interpretable can help create artificial intelligence models for designing healthy diets that meet specific nutritional requirements.

ML is how computers learn to do specific things without being specifically programmed. This happens through algorithms, sets of rules a computer follows to reach a goal: prediction, classification, or clustering (35). A subfield of artificial intelligence, supervised machine learning algorithms can learn from training data to develop a function that can model the relation between input variables (e.g., nutrients) and an outputs variable (e.g., classification into groups, equivalent portion) (36).

Therefore, we generate an algorithm using artificial neural networks to classify and calculate the equivalent portion. In the present study, once the tool has been created to compare the similarity between foods, we obtained the interpretability of each of the weights by using the flexibility of the SKM model to make a simple tool for weighting variables to predict the group category. Subsequently, using the vector formulation and the similarity equation, the foods with the most significant similarity with the target food were found within the database, and the equivalence factor of each one was calculated. This equivalence would be more exact in terms of nutrients if the factors of more than two foods could be optimized to reach a single target,


(9) C=αA+βB.

Where the foods A,B , and C belong to the same category with a previous classification.

The main reason for the idea of use of Spherical K-means, SKM, is the implementation of centroids and the cosine similarity. In this case the centroids were obtained as the means of the nutrients in each group from the initially defined set.

As observed, the performance of SKM model and even neural networks was not as high as the performance of XGBoost and Random Forest classifiers. However, the objective of these exchangeable lists is to provide an equivalent food, so it is proposed to omit the classification step since the nature of these algorithms allow little interpretability. This opens the alternative of selecting an equivalent food not from a group previously classified by machine learning, but with a completely interpretable algorithm such as cosine similarity for comparing foods.

On the other hand, multilayer perceptron (MLP) is a complement to feed forward the neural network. MLP consists of three layers: input, output (Food groups in our case), and hidden layer. Being the hidden layers, the ones that carry out the computational work of the multiple perceptrons. MLP is usually used for recognition, approximation, prediction, and pattern classification, which is used in our case. However, the information it has received to be trained is limited. In effect, it does not have the food’s origin so it can make an incorrect classification, but it was evident to humans.

FELs must be updated to introduce new foods or adapt them to specific countries or populations (4) since one of the challenges of the FELs is the scarcity of regional food and the need for up-to-updating. Using up to date FELs with local/regional foods will allow the designed diet to gain greater acceptance with a better chance of successful implementation and avoid adherence-related issues due to foods being limited to the food exchange list (37, 38). The model proposed in this study can classify and calculate the equivalent portion of a single or a list of foods. Due to the similarity formula, our model has enough flexibility to quickly compare a complete set of foods from a previously classified category. Therefore, it offers a helpful, agile, and versatile tool for the dietitian and the patient. Also, our model uses normalized input data, often used in neural networks, to speed up the training and improve the neural networks (39), thus allowing, even with a 3% error in the prediction of the food category, to give us a group of foods that offer a similar nutritional composition.

On the other hand, FELs should not only focus on macronutrients and the energy content of food; they should also consider the content of other nutrients such as sodium, added sugars, and cholesterol, among others (40). These nutrients are relevant because high intakes of sodium, cholesterol, and added sugar have been associated with an increased risk of developing hypertension, cardiovascular disease, obesity, insulin resistance, fatty liver, and type 2 diabetes (41). In addition to being a tool for designing eating plans, the SMAE indicates when the contribution of a nutrient in an equivalent portion is considered high in sodium, cholesterol, or sugar (29). It has been observed that a low-sodium diet prepared using an exchange list was more effective than the one designed using food composition tables (4).

Interestingly, the algorithm’s prediction had the worst performance during the validation with the legume group, and the second one with the worst performance during the training was the sugar with the fat groups. This could be because the characteristics of nutrients are very similar between each food analyzed, and this means that the neural network needs to have adequate training and that at the time of doing the test, it could perform better.

Finally, calculating the similarity coefficient, which is a much more flexible method compared with others (42), in the dimensionless space (43) results in the closeness in this space but not in the nutritional component space; working in this space has the advantage of eliminating the dependence on units, such as kcal, g, and mg, being able to calculate a very close approximation to what is expected and not the nearest position.

Ultimately, more is needed to have robust FELs generated with ML; it is also necessary that the information be transmitted in a didactic way to the patients. Bawadi et al. points out that using a human-friendly food exchange list can be implemented for people with low literacy, as it is based on visual techniques (32).

Is necessary to mention that this technique that is proposed in this manuscript has limitations. Being one of the most important that has to do with the database, which could have nutritional elements that are not known or have not been measured in new foods, this could cause a predisposition in the classification of the food. Although is a problem, it is not something common to see eating processed foods, which are regularly very well studied from the nutritional point of view. Something interesting that was developed in this work is the matter of dimensioning and normalizing the data, although it is additional process that takes a little more time, the advantage lies in comparing the nutritional concentration and not in distances for being a vector.

In conclusion, machine learning is a promising method for automation in generating FELs. This study provides evidence of a large-scale, accurate real-time processing algorithm that can be useful for designing meal plans tailored to the foods consumed by the population. Our model allowed us not only to distinguish and classify foods within a group or subgroup but also to perform the calculation of an equivalent food. As a neural network, this model could be trained with other food bases and thus improve its predictive capacity. Although the performance of the SKM model was lower compared to other types of classifiers, our model allows selecting an equivalent food not from a group previously classified by machine learning but with a fully interpretable algorithm such as cosine similarity for comparing food.

## Data availability statement

The datasets presented in this study can be found in online repositories. The names of the repository/repositories and accession number(s) can be found below: https://github.com/jovanidhh/SMAE-NN/tree/main.

## Author contributions

DH-H: formal analysis, data curation, and writing—original draft preparation. AP-L: investigation, methodology, and writing—review. BP-G and GM-L: conceptualization, methodology, and writing—review and editing. All authors contributed to the article and approved the submitted version.

## Conflict of interest

The authors declare that the research was conducted in the absence of any commercial or financial relationships that could be construed as a potential conflict of interest.

## Publisher’s note

All claims expressed in this article are solely those of the authors and do not necessarily represent those of their affiliated organizations, or those of the publisher, the editors and the reviewers. Any product that may be evaluated in this article, or claim that may be made by its manufacturer, is not guaranteed or endorsed by the publisher.
